# Meeting the challenges of recruitment to multicentre, community-based, lifestyle-change trials: a case study of the BeWEL trial

**DOI:** 10.1186/1745-6215-14-436

**Published:** 2013-12-18

**Authors:** Shaun Treweek, Erna Wilkie, Angela M Craigie, Stephen Caswell, Joyce Thompson, Robert JC Steele, Martine Stead, Annie S Anderson

**Affiliations:** 1 Health Services Research Unit, University of Aberdeen, Health Sciences Building, Foresterhill, Aberdeen AB25 2ZD, UK; 2 Tayside Clinical Trials Unit, University of Dundee, Ninewells Hospital & Medical School, George Pirie Way, Dundee DD1 9SY, UK; 3 Centre for Research into Cancer Prevention and Screening, Division of Cancer Research, Medical Research Institute, Level 7, Mailbox 7, University of Dundee, Ninewells Hospital and Medical School, Dundee DD1 9SY, UK; 4 Directorate of Public Health, Dundee NHS, Tayside, UK; 5 Institute for Social Marketing, University of Stirling and the Open University, Stirling FK9 4LA, UK

**Keywords:** Colorectal cancer, Multicentre trial, Recruitment

## Abstract

**Background:**

Recruiting participants to multicentre, community-based trials is a challenge. This case study describes how this challenge was met for the BeWEL trial, which evaluated the impact of a diet and physical activity intervention on body weight in people who had had pre-cancerous bowel polyps.

**Methods:**

The BeWEL trial was a community-based trial, involving centres linked to the Scottish National Health Service (NHS) colorectal cancer screening programme. BeWEL had a recruitment target of 316 and its primary recruitment route was the colonoscopy clinics of the Scottish Bowel Screening Programme.

**Results:**

BeWEL exceeded its recruitment target but needed a 6-month no-cost extension from the funder to achieve this. The major causes of delay were lower consent rates (49% as opposed to 70% estimated from earlier work), the time taken for NHS research and development department approvals and the inclusion of two additional sites to increase recruitment, for which there were substantial bureaucratic delays. A range of specific interventions to increase recruitment, for example, telephone reminders and a shorter participant information leaflet, helped to increase the proportion of eligible individuals consenting and being randomized.

**Conclusions:**

Recruitment to multicentre trials is a challenge but can be successfully achieved with a committed team. In a UK context, NHS research and development approval can be a substantial source of delay. Investigators should be cautious when estimating consent rates. If consent rates are less than expected, qualitative analysis might be beneficial, to try and identify the reason. Finally, investigators should select trial sites on the basis of a formal assessment of a site’s past performance and the likelihood of success in the trial being planned.

**Trial registration:**

Current Controlled Trials ISRCTN53033856

## Background

Randomized controlled trials (RCTs) are the gold standard for the evaluation of the effectiveness and safety of healthcare interventions, particularly because they protect against selection bias [1]. However, recruiting health professionals and patients to RCTs can be extremely difficult: studies of recruitment suggest that at least 45% of studies fail to achieve their recruitment targets, although those involving a clinical trials unit do somewhat better, with 65% (20/31) of trials recruiting to target [2,3]. This may result in an underpowered trial, which, in turn, may lead to non-significant results that nevertheless do not rule out the possibility of important benefits. Recruitment failings increase the risk that an effective intervention will be abandoned before its true value is appreciated, or can lead to delays in demonstrating the benefits of an intervention while further trials are conducted. Poor recruitment (and retention) may also lead to a trial being extended, increasing costs. Investigators use many interventions to improve recruitment [4-6] but evidence regarding the likely effect of these interventions is often unclear.

Community-based studies face particular challenges because of the dispersed nature of both the recipients of services and the professionals delivering the services [7]. Recruitment efforts can be hampered by actual or perceived demands of transportation, unfamiliarity with study sites for appointments, and inflexible appointment times within working hours. The Cochrane review of interventions to improve recruitment has a planned subgroup analysis comparing studies by setting (for example, community versus secondary care recruitment) but has not found enough community and primary care studies to include in the analysis, despite including a total of 45 studies [5]. More rigorous evaluations of recruitment interventions are needed, especially in community and primary care.

The BeWEL study was a multicentre RCT evaluating the impact of a diet and physical activity intervention on body weight in people who have had pre-cancerous bowel polyps [8]. The relationship between diet, physical activity, excess weight and increased risk of colorectal cancer (and other chronic disease) is well described [9] and the National Health Service (NHS) colorectal cancer screening programme offers a timely opportunity to offer intervention support. The aim was to recruit 316 eligible participants over a period of 12 months. However, obtaining the necessary research governance and ethical approval went more slowly than expected, which quickly led to a shorter time frame to meet the recruitment target. This paper aims to critically review the methods employed to overcome this challenge and so provide insight into the implications for future trials.

## Methods

BeWEL’s sample size was calculated on the basis of a clinically important weight loss at 12 months of 7%, which at 80% power meant that 133 participants would be required to complete each arm of the study. The target of 7% weight loss at 12 months was chosen as this has been found to be effective for diabetes prevention [10]. Allowing for a dropout rate of 16%, as seen in the similar Bowel Health to Better Health (BHBH) study [11], meant that 158 participants were required for each arm, giving a recruitment target of 316. Individuals were eligible for the trial if they were aged 50 to 74 years with a BMI greater than 25 kg/m^2^ and had been found to have benign adenomas after a screening colonoscopy done as part of the Scottish Bowel Screening Programme (http://www.bowelscreening.scot.nhs.uk). Individuals also needed to have been physically able to undertake the trial’s exercise requirements and to be able to provide informed consent. Individuals were not eligible for the trial if they had a normal colonoscopy, malignant lesion or insulin dependent diabetes.

The participants for BeWEL were recruited through the colonoscopy clinics of the Scottish Bowel Screening Programme. The planned recruitment strategy [8] was as follows:

1. A brief letter about BeWEL from the colorectal cancer surgeon, enclosed with the screening results, endorsing the study and encouraging the recipient to read an information sheet that would be sent by the study team within two weeks.

2. An information leaflet, letter of invitation, reply slip and pre-paid envelope was then sent by the study team.

3. Those indicating that they would like to take part were screened for eligibility over the telephone by a research nurse. Eligible individuals were then invited to attend a study centre to provide consent and undergo baseline measures.

4. Non-responders were sent a reminder after two weeks, which included the information leaflet, letter of invitation, reply slip and pre-paid envelope.

Based on this plan and an estimated positive response rate of 70%, based on a previous study of diet and physical activity [11], three colonoscopy clinics were recruited to take part in the trial, one each in the Tayside, Forth Valley, and Ayrshire and Arran regions of Scotland. Monthly trial management group meetings monitored recruitment using site-specific CONSORT diagrams, as well as a CONSORT diagram for the trial as a whole.

### Approvals

BeWEL was approved by the Tayside Committee on Medical Research Ethics (Committee B), REC reference 10/S1402/34 and received research and development approval from NHS Ayrshire and Arran, NHS Forth Valley, NHS Greater Glasgow and Clyde and NHS Tayside.

## Results

**Figure 1:**
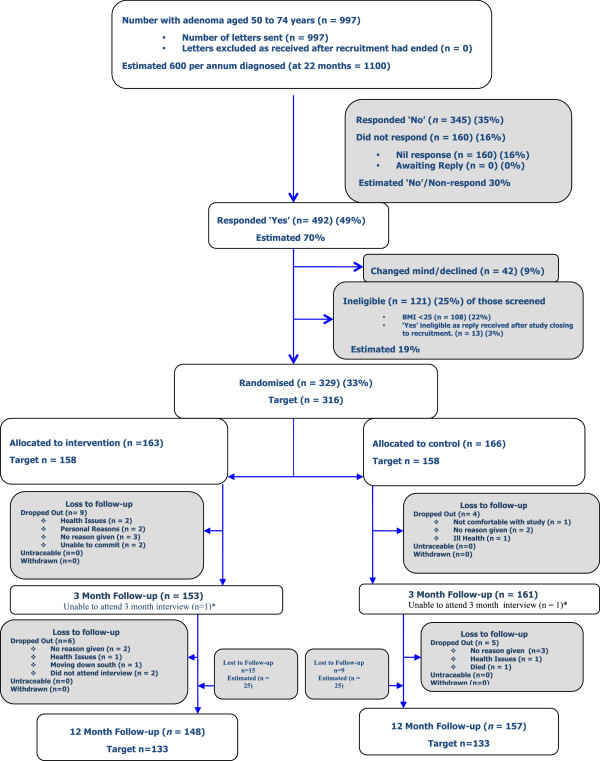
**Participant flow through the trial.** Where a number is marked as an estimate, this is the number we used pre-trial for planning. * Participants attended the 12-month interview.

A diagram of participant flow is given in Figure 1. A total of 997 letters of invitation were sent between November 2010 and April 2012 (17 months) to individuals who had undergone colonoscopy following a positive faecal occult blood test as part of the Scottish Bowel Screening Programme, had a diagnosis of adenoma confirmed by histopathology and were aged 50 to 74 years. To the best of our knowledge, these were all the patients screened in the participating Health Boards for whom an adenoma was detected. Of these, 492 replied positively (49%) but 108 (22%) were ineligible as their BMI was less than 25 kg/m^2^, 42 (9%) declined to proceed after receiving detailed information of the requirements and 13 (3%) replied after the recruitment period had ended. Thus, 329 patients went on to be randomized (33%), exceeding the target recruitment of 316. Retention was higher than estimated, leading to a larger pool for analysis (305) than was required in the sample size calculation (266) (Table 1).

**Table 1:** Estimates used in planning recruitment and the numbers actually achieved in the trial

**Recruitment assumption**	**Estimate**	**Actual**
Loss to follow-up	16%^a^	7%
Consent rate	70%^a^	49%
Recruitment target	316	329

^a^Data from [11].

Although the recruitment target was met, this was not achieved without modifying the original recruitment plan. The strategies introduced are listed in Table 2. Upon the initial delayed start to recruitment, the first modification was made, which was to approach new sites for recruitment and begin the approvals process. By month three, the positive response and randomization rates were 31% and 17%, respectively, indicating a need for further efforts to improve recruitment. Strategies 2 to 8 were then introduced.

**Table 2:** Recruitment strategies employed in the BeWEL trial

**Strategy number**	**Strategy**	**Date started (month number)**
1	New sites approached for study inclusion.	November 2010 (month 1)
2	Research nurse telephones non-responders (suggested by [4,5]).	January 2011 (month 3)
3	BMI cut-off at telephone screening reduced to 24 kg/m^2^ to avoid excluding participants who had underestimated their BMI. Such participants were invited to visit the research centre to have their eligibility confirmed.	January 2011 (month 3)
4	Frequency of visits by the trial manager to research nurses and on-site NHS staff increased to monthly.	May 2011 (month 7)
5	Brief participant information leaflet introduced and printed on high-quality paper with NHS logos. This was sent with the invitation letter in hand-written envelopes (suggested by [12,13]).	June 2011 (month 8)
6	The local consultants’ names and their endorsement of the study were added to the invitation letter.	June 2011 (month 8)
7	Eligible participants unable to travel to a study site for assessments were offered home visits.	July 2011 (month 9)
8	Letters of congratulation were sent to sites for good recruitment.	November 2011 (month 13)

### Strategy 1: New sites approached for study inclusion

Research staff in the UK who do not have contracts with the NHS need a document called a ‘research passport’ before they can contact NHS patients or access their data (see http://www.ukcrc.org/regulationgovernance/researchpassport/). Obtaining these documents took longer than anticipated, meaning that recruitment to BeWEL at all sites began three months late. Therefore, in November 2010, an initial approach was made to Gartnavel General Hospital, NHS Greater Glasgow and Clyde (GG&C), to discuss the BeWEL study and seek approvals. By March 2011, a further site in Fife had also been approached but the GG&C site was still awaiting NHS research and development (R&D) approvals. In April 2011, the Trial Steering Committee met and suggested that the site in Fife be kept on hold and the study be extended for a short period to meet its recruitment target rather than having to go through the time-consuming permissions process. Subsequently, an additional GG&C site (Victoria Infirmary) became involved, through local consultant interest. Both additional sites were positive and the process of gaining ethical approval was smooth. The process of gaining R&D approvals and issuing research passports, as well as delays caused through clinicians needing to provide paperwork, meant that it took seven months and considerable effort from the trial team to gain all the relevant permissions for Gartnavel General Hospital (submitted November 2010 and approved July 2011). The second of the two sites, Victoria Infirmary, agreed to take part in September 2011 but obtaining permissions took until 24 January 2012, with just four months recruitment time remaining. By the end of recruitment, only five participants were recruited through the two Glasgow sites.

### Strategy 2: Research nurse telephones non-responders

There is high-quality evidence that telephone reminders to non-responders increase trial recruitment [4,5] and telephone reminders were added to the protocol after the first three months of recruitment. Two weeks after the reminder invitations were sent out, the research nurses telephoned individuals who had not responded, up to a maximum of three times over a two-week period. This required a change to the invitation letter to make it clear that the trial team would telephone non-responders, and a substantial amendment to be submitted to the ethics committee for approval (submitted 21 December 2010 and approved 24 January 2011). Telephone reminders were time consuming but led to the recruitment of an additional 14 participants (Table 3).

**Table 3:** Recruitment return of telephone reminders to non-responders to a postal reminder invitation

**Site identifier: health board**	**Number sent reminder letter**	**Number of non-responders to reminder letter (%)**	**Number of non-responders telephoned (%)**	**Number not responding to any calls (%)**	**Number recruited after call (%)**
Site 1: Tayside	141	70 (50)	27 (39)	4 (15)	0 (0)
Site 2: Forth valley	103	52 (50)	40 (77)	18 (45)	5 (13)
Site 3: Ayrshire & Arran*	67	33 (49)	28 (85)	2 (7)	9 (32)
Site 4: Greater Glasgow	7	7 (100)	3 (44)	0 (0)	0 (0)
Site 5: Greater Glasgow	2	1 (50)	0 (0)	0 (0)	0 (0)
**Total**	**320**	**163 (51)**	**98 (60)**	**24 (24)**	**14 (14)**

*Owing to a misunderstanding, Ayrshire and Arran only started to follow the telephone reminder protocol in July 2011, not January 2011, as at the other sites.

### Strategy 3: BMI cut-off at telephone screening reduced to 24 kg/m^2^

It is widely documented that there is a tendency for height to be overestimated and weight to be underestimated, such that self-reported BMI is often underestimated, especially in men [14]. The trial management group therefore agreed that potential participants who self-reported their BMI to be 24 to 25 kg/m^2^ be invited into the study centre to have their eligibility checked. This resulted in screening an additional 15 individuals, of whom ten were eligible and all were recruited.

### Strategy 4: Frequency of visits by the trial manager to research nurses and on-site NHS staff increased to monthly

The trial manager was in daily email or telephone contact with site research nurses to offer support as necessary. The trial management group initiated monthly visits to sites by the trial manager to offer face-to-face support to research teams and to maintain the visibility of the trial at the sites. In total, there were 17 visits to Ayrshire and Arran, 15 to Forth Valley and six to GG&C. The situation for Tayside was somewhat different, since the trial manager was based at the same hospital and there was therefore very regular contact between the trial manager and research nurse at this site. Although the support and endorsement of consultants at each site was essential, it was also important to establish good working relationships with all referring and administrative staff. An administrative assistant was also employed to support research nurses and lifestyle counsellors with the day-to-day running of the trial. Although supporting these relationships was considered essential for the success of BeWEL, there is very little published evidence regarding their effect on recruitment [4-6]. A single study has evaluated the effect of site initiation visits and found that they did not increase recruitment [15]. That study’s host trial was terminated early and the effect of repeat visits on recruitment could not be studied.

### Strategy 5: Brief participant information leaflet introduced and printed on high-quality paper with NHS logos

The full participant information leaflet (PIL) was an eight-page document covering all aspects of the trial, including mandatory sections that were required for ethical approval. Although the evidence for trial recruitment benefit (or harm) for short versus long PILs is unclear [4,5], there is evidence that shorter questionnaires lead to higher response rates [12,13]. A brief-two page PIL was also prepared to be sent two weeks after the initial invitation letter. In addition, the NHS logo was added to both the brief and full PILs, to take advantage of positive feelings about the NHS screening service that were reported during BeWEL’s formative work. Finally, “Please note this study does not involve any further bowel examination,” was added to the brief PIL, as a number of participants had asked whether they had to have another colonoscopy as part of the study. Ethical approval was obtained to introduce the brief PIL on the understanding that the full leaflet would be provided to potential participants before consent was obtained. Introducing the brief PIL required submission of a substantial amendment to the ethics committee for approval (submitted 23 June 2011 and approved 29 June 2011).

### Strategy 6: The local consultants’ names and their endorsement of the study were added to the invitation letter

The invitation letter was amended to show the consultant’s name and his endorsement of the study. This decision was based on a belief that potential participants would be more likely to respond to a letter signed by someone they recognised. There is no compelling evidence for such an effect [13], although the possibility of a small benefit is not ruled out completely. The strategy was, however, simple and cheap to implement. This required a substantial amendment to be submitted to the ethics committee for approval (submitted 23 June 2011 and approved 29 June 2011).

### Strategy 7: Eligible participants unable to travel to a study site for assessments were offered home visits

Some participants in Ayrshire and Arran and in Tayside, both large and rural areas, found it difficult to travel to a study site for assessments. To address this, the protocol was amended to include the possibility of home visits by research nurses for participants in any of the areas taking part in BeWEL. Ten participants opted to have home assessments, all from Tayside. Adding home visits required a substantial amendment to be submitted to the ethics committee for approval (submitted 29 June 2011 and approved 14 July 2011).

### Strategy 8: Letters of congratulation to sites for good recruitment

A total of six letters of congratulation were sent out to participating sites (three to Tayside, three to Ayrshire and Arran) between January and June 2012. The sites were commended for their continued support for the study and told how many of their referrals were eligible and had given signed consent to participant in the BeWEL study.

### Combined impact of the additional recruitment strategies on accrual

**Figure 2:**
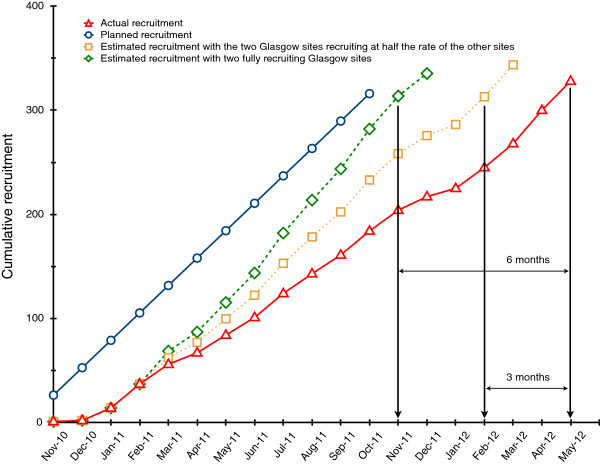
**Planned, actual and estimated total monthly recruitment.** The estimated monthly recruitment rate is based on two scenarios. Scenario 1 (dotted line): the two Glasgow sites had a *combined* monthly recruitment rate equivalent to the average of the monthly rates at the three other sites. Scenario 2 (dashed line): the two Glasgow sites each recruited at a similar rate to the other three sites.

**Figure 3:**
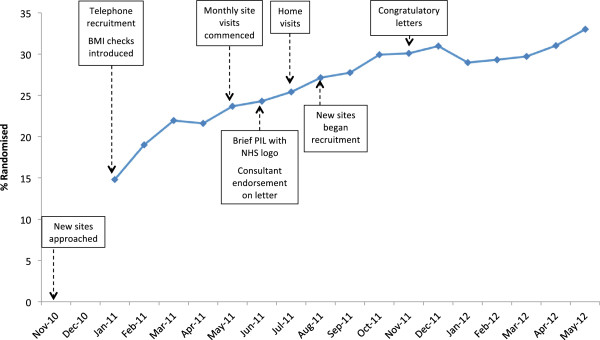
The proportion of eligible individuals who were randomized each month.

The first and most lengthy recruitment strategy instigated was the addition of two further sites. Figure 2 shows the actual cumulative monthly recruitment along with planned recruitment and estimated monthly recruitment if the two Glasgow sites had begun recruiting in March 2011 (four months after the start of the trial) under two scenarios. In the first scenario (dotted line), the two Glasgow sites have a *combined* recruitment equivalent to the average of the monthly rates at the three other sites. In the second scenario (dashed line), each of the Glasgow sites recruits at a rate equivalent to the average of the monthly rates at the other three sites (see Table 4). In the former scenario, the BeWEL trial would have reached its recruitment target around three months earlier, in February 2012. In the latter scenario, the recruitment target would have been reached six months earlier, in November 2011, or just one month behind the original schedule. Finally, the percentage of eligible individuals who consented to take part and went on to be randomized increased steadily throughout the trial from 17% in Jan 2011 to 33% in May 2012, when the trial reached its recruitment target (Figure 3).

**Table 4:** Monthly recruitment figures by site, including estimated monthly recruitment at Greater Glasgow under two scenarios

**Month**	**Ayrshire & Arran**	**Forth valley**	**Tayside**	**Actual, greater Glasgow**	**Estimated, Greater glasgow (scenario 1)**	**Estimated, greater Glasgow (scenario 2)**	**Actual total monthly recruitment**	**Estimated total monthly recruitment (scenario 1)**	**Estimated total monthly recruitment (scenario 2)**
November 2010	0	0	1	0	0	0	1	1	1
December 2010	0	1	0	0	0	0	1	1	1
January 2011	2	7	3	0	0	0	12	12	12
February 2011	6	11	6	0	0	0	23	23	23
March 2011	3	6	10	0	6	13	19	25	32
April 2011	3	7	1	0	4	7	11	15	18
May 2011	3	7	7	0	6	11	17	23	28
June 2011	5	4	8	0	6	11	17	23	28
July 2011	9	6	8	0	8	15	23	31	38
August 2011	9	5	5	0	6	13	19	25	32
September 2011	7	8	3	0	6	12	18	24	30
October 2011	11	5	7	0	8	15	23	31	38
November 2011	6	7	6	1	6	13	20	25	32
December 2011	9	2	21	0	4	8	132	17	
January 2012	3	1	4	0	3	5	8	11	
February 2012	7	4	9	0	7	13	20	27	
March 2012	7	6	10	0	8	15	23		
April 2012	13	10	9	0	11	21	32		
May 2012	18	3	4	4	8	17	29		
**Total**							**329**	**314**	**313**

Scenario 1, the two Glasgow sites on board in March 2011 and have a combined recruitment equivalent to the average of the monthly rates at the three other sites; scenario 2, the two Glasgow sites on board in March 2011 and recruit at a rate equivalent to the average of the monthly rates at the other three sites. In scenario 1, BeWEL would have reached its target about three months earlier; in scenario 2, the target would have been met about six months earlier.

## Discussion

The BeWEL trial met its recruitment target. However, despite close monitoring and considerable resources and effort being targeted at recruitment, the trial still required a six month no-cost extension from the funder to meet this target. Although the funder provided no additional funding, extending the trial by six months was possible because of flexibility shown by contracted staff and because core-funded and other departmental staff contributed more time. By three months into the trial, it was clear that recruitment was not going as planned and a series of interventions to increase recruitment were implemented, including interventions with evidence of benefit from systematic reviews (for example, telephone reminders to non-respondents [4,5]). Whilst these probably helped, as demonstrated in Figure 3, which shows a steady increase in the proportion of eligible individuals consenting and being randomized, there was no magic-bullet recruitment intervention that led to a step change in recruitment rates. Some interventions required substantial effort, such as repeated telephone attempts to contact participants and visiting remote rural locations. It is also clear that, as others have reported [16], recruitment in the first couple of months or so is indicative of later recruitment unless action is taken. To have assumed that slow recruitment was merely trial growing pains rather than a problem to be dealt with immediately would have been a mistake.

There are essentially two issues at the heart of BeWEL’s recruitment challenges:

1. Estimating the number of potentially eligible participants who will agree to take part;

2. Introducing extra recruitment sites.

### Estimating the number of potentially eligible participants who will agree to take part

The estimate of 200 patients diagnosed each year at the three original sites turned out to be accurate but the proportion who were eligible was 75%, not 81%, as estimated. This relatively small difference was then compounded by eligible participants who changed their mind after initially saying yes (42, or 9%) to participation, which was not expected.

A greater problem though, was the consent rate, which was 49%, not the estimated 70%. Formative qualitative research was conducted to help check the acceptability of the intervention concept and to refine methods before recruitment started [17]. This gave insight into how the initial approach to patients could be improved; for example, that colorectal cancer health professionals act as advocates for the study and repeat its endorsement by the lead clinician, suggestions that were incorporated into the recruitment strategy for BeWEL. One important finding from the formative research was that patients had little understanding of the potential link between adenoma risk and their own health behaviour, and consequently struggled to see the relevance of an invitation to participate in a lifestyle-change study. This was reinforced by the tendency of health professionals during and after adenoma treatment to adopt a reassuring tone that downplayed risk. The ‘all-clear’ messages that patients picked up from written and verbal communication after their adenoma operation implied a ‘clean bill of health’ and indicated that there was nothing about their lifestyle requiring modification. Although efforts were made in the trial recruitment process to address this problem by making clearer the potential links between adenoma risk and lifestyle behaviour, it is possible that the link was still not sufficiently salient or believable for some patients, and that this may have contributed to reluctance to participate in the study.

The estimated consent rate of 70% was based on the BHBH study [11], a trial similar to BeWEL. The BHBH study reported two consent rates: an overall rate of 51% and an ‘initial’ rate of 68%; the latter rate was the rate seen before a second Dundee-based trial started recruiting from the same patient pool. The 68% rate seemed to be a reasonable choice for BeWEL, given the clear link between the fall in the BHBH study consent rate and the start of recruitment by the second trial. As we found later, the overall BHBH consent rate would have been a better bet. Additionally, although the BHBH study was similar to BeWEL there were two key differences. The first was that the BeWEL intervention placed more demands on patients, requiring a 12-month commitment from participants rather than three. The second was that weight management was included in BeWEL but not in the earlier trial, which again might have deterred some from participating. Although most of the BeWEL participants who were interviewed at the end of the program found the 12-month duration and inclusion of weight management acceptable and not too onerous or intrusive, these were of course patients who had agreed to participate; we do not know how many were put off coming forward in the first place by the perceived demands of participation in the study.

There are a couple of key lessons in this experience. Firstly, how should investigators estimate consent rates? One simple approach would be for investigators to estimate no more than 50% unless they have experience of higher consent from several studies in the same population being recruited in the same setting. This appears rather arbitrary but a study of recruitment in 207 breast cancer trials calculated the number needed to recruit one additional participant for the 69 trials that provided sufficient information to do the calculation and the result was remarkably consistent, with a median of two individuals being approached for every person recruited [18]. Gross *et al*. [19] found a median of 1.8 (range, 1 to 68) for their study of 172 trials, whereas Toerien *et al*. [20], in their study of 133 trials, found that investigators assessed a median of 230% of their target number. A consent rate estimate of 50% is perhaps a reasonable rule of thumb in the absence of compelling evidence upon which to base it. More compelling evidence would comprise data from two or more studies that have recruited the same population, in the same setting, using the same sort of staff, for the same sort of intervention and all within recent history. Even with these data, investigators would need to make evidence-informed, judgement-based decisions about the similarity between earlier recruitment contexts and their own. It would be possible to put confidence intervals around consent rates from other studies, including pooled estimates, but it is context, not statistical uncertainty, that is likely to be the main driver of variability in consent rates. It is far from clear that taking the lower bound of the confidence interval would provide more reassurance than would be the case if investigators (ourselves included) were simply more conservative when estimating consent rates, and many other parameters besides. The best approach to consent rate planning remains in-context pilot work prior to the full-scale trial.

The second lesson was that once it became apparent that fewer patients were consenting to take part, one additional strategy would have been to conduct a second stage of qualitative research. This need not have been a major exercise, as one or two focus groups or a small number of individual interviews would have sufficed. This second stage could have focused on exploring the views of those patients who expressed an initial interest but then did not follow through, to see whether any of the reasons for reluctance were amenable to action. This kind of research exercise has been used to explore how patients interpret and respond to informed consent materials provided in clinical trials [21]. ‘Consumer research’ of this sort could play a valuable role throughout the development and implementation of an intervention in remedying problems as they occur. Indeed, given the commonplace nature of trial process problems, investigators would do well to build in the possibility of adding rapid, response-mode qualitative work to their initial ethical and other approval submissions.

#### Bringing on extra sites

There were 13 months between the trial management team’s first discussion with a Glasgow site and the first Glasgow recruit. At the end of the trial, the two sites together had been able to recruit only five participants. Conjecture as to what might have been is, perhaps, of limited utility but had the two extra sites both recruited at a similar rate to the other sites (Scenario 2 in Figure 2 and Table 4), then the trial would have met its target only one month behind the original recruitment schedule. In the three original sites, considerable negotiation had been undertaken well before the funding bid had been submitted, and again after the funding award announced, highlighting the time required to match sites to study requirements. Thus, by the time the study started, the preparatory work at each site had been undertaken. This was not the case in the two Glasgow sites, where many months were taken up as a consequence of NHS R&D departmental work practices being different from other sites, a situation made worse by the loss of the trial manager to take up another post during the latter stage of funding negotiations. Moreover, the anticipated time for recruitment from these two sites was not fully factored in to the revised recruitment plan.

This experience points towards a number of suggestions linked to site selection:

• Identify more sites than are expected based on pre-trial assumptions as an insurance policy against those assumptions proving incorrect. The number of extra sites will depend on how confident investigators are about their pre-trial assumptions. If all, or some of the approvals for these sites can be obtained up-front (that is, before they are actually needed), so much the better.

• Formally assess all sites for suitability for the trial. The trial team should use a checklist of key features of a site that they believe are essential for successful participation. The team could develop its own checklist, or modify an existing one (for examples, see Warden *et al.*[22]).

• Sites that do not meet the requirements listed on the checklist should be reviewed to determine whether measures could be put in place by the trial team to support the site in meeting the checklist criteria. If not, the site should not be considered for the trial.

Clearly, no site is ideal for all trials but there is growing agreement that sites should be selected based on a formal assessment of their past performance and the likelihood of success in the trial being planned [22-24]. As Shah pointed out in a recent roundtable discussion of heart failure trials [23]:

‘*There is a peculiar paradox that exists in trial execution - we perform clinical trials to generate evidence to improve patient outcomes; however, we conduct clinical trials like anecdotal medicine: (1) we do what we think works; (2) we rely on experience and judgement and (3) limited data to support best practices.*’

What features of a site might predict its future performance in a given trial is worthy of more research but some features that may be relevant [22-24] include:

• Previous experience with multicentre trials;

• Familiarity of operating a trial protocol and the closeness of the trial protocol to the clinical procedures currently in place at the site;

• Familiarity with the local approvals process;

• Previous recruitment performance;

• Case mix and access to eligible participants;

• Availability of resources such as research nurses, study coordinators, research pharmacists, administrators;

• Lack of competing demands that would hinder the site’s ability to fully engage with the trial.

Future research could focus on systematically reviewing the trial management literature for studies in order to evaluate formal site-selection methods and develop prediction rules and metrics that can be used for site selection.

## Conclusions

Recruitment plans rarely survive contact with actual participants [2]. Though challenging, recruitment to multicentre trials can be successfully achieved with a committed team. In a UK context, NHS R&D management approval can be a substantial source of delay, whilst obtaining ethical approval is a much smoother process. Investigators should be cautious when estimating consent rates and it may be beneficial to do qualitative work during the trial if consent rates are less than expected, to try and identify the reason. Finally, investigators should select trial sites on the basis of a formal assessment of a site’s past performance and the likelihood of success in the trial being planned.

## Abbreviations

BHBH: Bowel health to better health; BMI: body mass index; GG&C: Great Glasgow and Clyde; NHS: National Health Service; PIL: participant information leaflet; R&D: research and development; RCT: randomized controlled trial.

## Competing interests

The authors declare that they have no competing interests.

## Authors’ contributions

ST, ASA, RS, AC, SC, JT, MS and EW all contributed to the design of the study and to the interpretation of its results. ST wrote the first draft of the paper and all authors commented on it and subsequent drafts. All authors approved the final version. All authors meet the guidelines for authorship of the International Committee of Medical Journal Editors.
